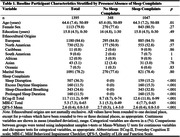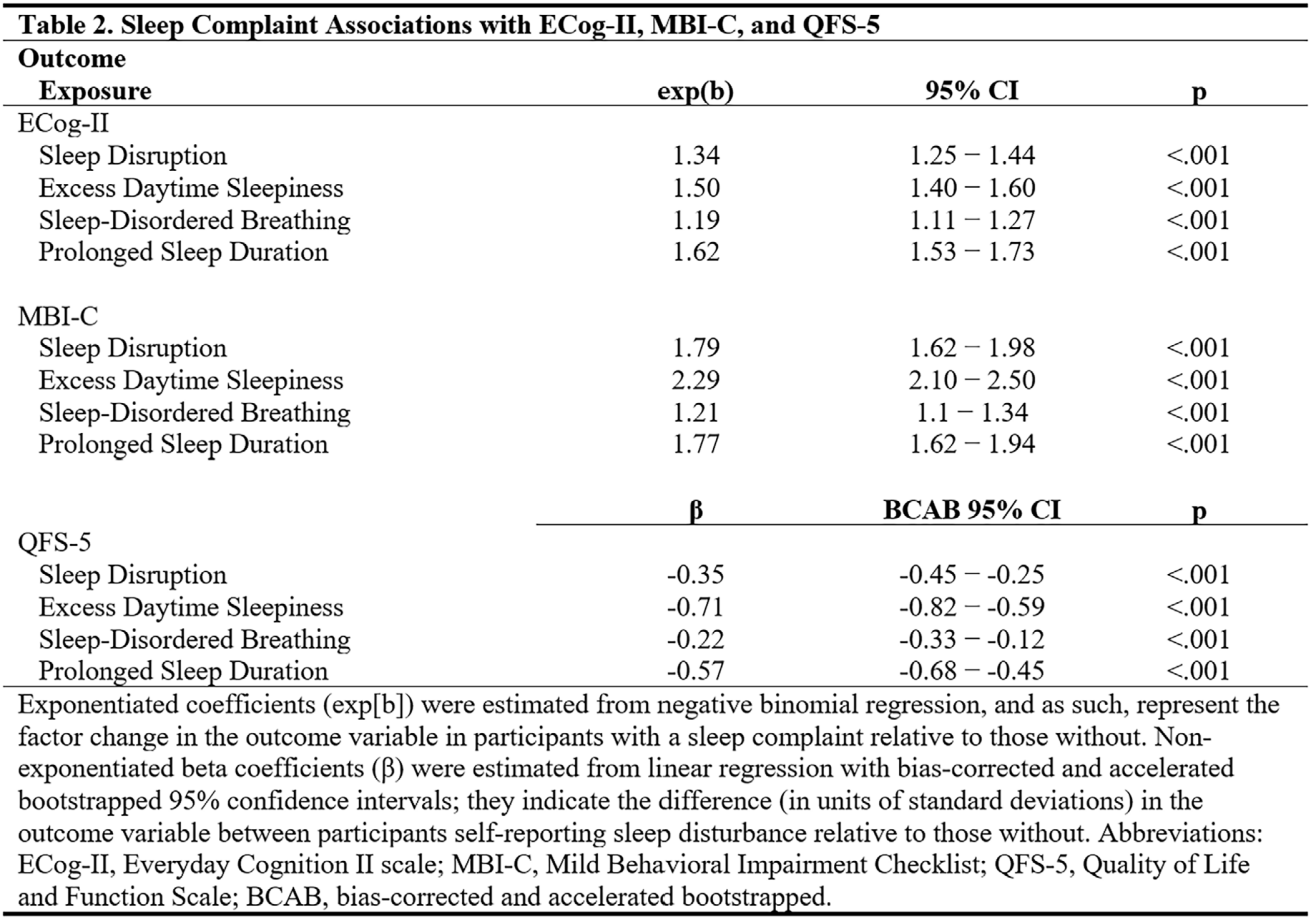# Cognitive, behavioral, and quality of life outcomes in cognitively unimpaired older persons with or without sleep complaints

**DOI:** 10.1002/alz.091648

**Published:** 2025-01-03

**Authors:** Dylan X. Guan, Andrew Beaudin, Eric E. Smith, Zahinoor Ismail

**Affiliations:** ^1^ Hotchkiss Brain Institute, University of Calgary, Calgary, AB Canada; ^2^ University of Calgary, Calgary, AB Canada; ^3^ Department of Clinical Neurosciences and Hotchkiss Brain Institute, University of Calgary, Calgary, AB Canada

## Abstract

**Background:**

Changes in sleep are common in older persons and have been linked to higher dementia risk. The link between sleep complaints and early risk markers of Alzheimer’s disease (AD), namely subjective changes in cognition and mild behavioral impairment (MBI), have not been fully explored. This study investigated associations between sleep complaints with cognitive and behavioral AD risk markers and quality of life (QoL) among cognitively unimpaired older persons. We hypothesized that older persons reporting sleep changes would also report more severe cognitive and behavioral changes, and poorer QoL.

**Method:**

Participants (n = 1395) were from the Canadian Platform for Research Online to Investigate Health, Quality of Life, Cognition, Behaviour, Function, and Caregiving in Aging (CAN‐PROTECT) study. Sleep complaints were self‐reported presence or absence of sleep disruption (symptoms of insomnia or sleep‐related movement disorders), daytime sleepiness, sleep‐disordered breathing, or prolonged sleep duration. Outcomes included the Everyday Cognition (ECog‐II) scale, Mild Behavioral Impairment Checklist (MBI‐C), and Quality of Life and Function (QFS‐5) scale. Age, sex, years of education, marital status, and ethnocultural origin were balanced across exposure groups using inverse probability of treatment weighting. Negative binomial and bootstrapped linear regressions were used, as appropriate, to model sleep complaint (exposure) associations with ECog‐II and MBI‐C total scores, and QFS‐5 mean scores (outcomes).

**Result:**

Participant characteristics are summarized in Table 1. Table 2 shows that all sleep complaints were associated with poorer everyday cognition, more severe MBI symptoms, and poorer QoL. However, excessive daytime sleepiness was generally the most strongly associated with the three outcomes with them having 1.50 (95%CI [1.40, 1.60]) and 2.29 (95%CI [2.10, 2.50]) times higher ECog‐II and MBI‐C score compared to participants without daytime sleepiness, and a 0.71 SD (95%CI [0.59, 0.82]) lower QFS‐5 mean score.

**Conclusion:**

Sleep complaints, even among cognitively unimpaired older persons, are linked to cognitive and behavioral risk markers of AD, as well as poorer QoL. These findings provide further evidence that sleep complaints represent modifiable risk factors or may reflect a prodrome for AD, and may contribute to overall disease burden.